# Using HLA-DR3-CBA/J Humanized Mice to Develop a Novel Genetic Model for Autoimmune Thyroiditis

**DOI:** 10.3390/genes17020170

**Published:** 2026-01-31

**Authors:** Aizhan Kozhakhmetova, Mihaela Stefan-Lifshitz, Olga Meshcheryakova, Yaron Tomer

**Affiliations:** Division of Endocrinology, Department of Medicine, The Fleischer Institute for Diabetes and Metabolism, Albert Einstein College of Medicine, 1300 Morris Park Ave, Bronx, NY 10461, USA

**Keywords:** autoimmune thyroiditis, mouse model, thyroglobulin, T cells, thyroid

## Abstract

Background: Experimental autoimmune thyroiditis is an important animal model for studying Hashimoto’s thyroiditis. Our aim was to develop the model using CBA/J-DR3 mice expressing human HLA-DR3, which is associated with autoimmune thyroiditis in humans, to better simulate human autoimmune thyroiditis. Such a humanized model can be used to test specific antigen therapies for autoimmune thyroiditis. Methods: CBA/J-DR3 mice were produced by back-crossing B6-DR3 mice to the CBA/J background. Female CBA/J-DR3 mice were immunized with human thyroglobulin (Tg) in complete Freund’s adjuvant on days 0 and 7. On day 21, mice were sacrificed, blood collected, spleen and thyroid harvested for analysis. Splenocytes were analyzed for T cell responses to Tg and its major T-cell epitope in human autoimmune thyroiditis, Tg.2098. Serum anti-thyroglobulin antibodies were measured by ELISA, and thyroid-stimulating hormone was measured using the Luminex assay. Thyroid histology and immunohistochemistry were examined. Results: Immunized CBA/J-DR3 mice showed significant T cell proliferation in response to Tg (stimulation index 3.4 ± 4.5) and Tg.2098 (1.5 ± 0.7). Anti-thyroglobulin antibody levels were elevated in immunized mice when compared to control mice (2.05 ± 0.75 vs. 0.15 ± 0.06, *p* < 0.0001). T cells demonstrated higher reactivity to thyroid antigens by enhanced production of pro-inflammatory cytokines. Thyroid immunohistochemistry revealed mild CD3-positive T-cell infiltration. Conclusions: This novel humanized CBA/J-DR3 mouse model of Hashimoto’s thyroiditis demonstrates key features of human autoimmune thyroiditis. The HLA-DR3 background and the immune response to Tg and Tg.2098 enhance translational relevance, making this a valuable model for studying thyroid disease pathogenesis and testing targeted immune-modifying therapies.

## 1. Introduction

Experimental autoimmune thyroiditis (EAT) is a widely used animal model for studying Hashimoto’s thyroiditis (HT), one of the most common organ-specific autoimmune diseases in humans, affecting 1–5% of the general population, predominantly women. Over the years, researchers have developed various EAT models to investigate the pathogenesis of autoimmune thyroid diseases (AITD) and test potential therapeutic interventions.

The classical EAT model, first described in 1956, involves immunizing susceptible mouse strains with thyroglobulin (Tg) emulsified in complete Freund’s adjuvant (CFA) [1,2,3]. This method reliably induces thyroid autoimmunity characterized by lymphocytic infiltration of the thyroid gland, production of anti-Tg antibodies (TgAb), and T cell responses against Tg. While this model has been instrumental in advancing our understanding of thyroid autoimmunity, it has limitations, particularly in studying the role of specific human Tg variants or domains in disease induction. Therefore, earlier we developed a more sophisticated EAT model to overcome some limitations of the classical EAT model. Our new model involved cDNA immunization, where plasmid DNA encoding full-length human Tg (Tg) is injected into skeletal muscle and followed with electroporation [2]. This method allowed for the presentation of non-iodinated Tg to the immune system and enabled genetic modifications of the Tg used in immunization. The cDNA immunization approach has shown promise in generating both humoral and T cell responses against Tg without the need for strong adjuvants like CFA [4].

Another innovative approach that we developed utilizes adenovirus vectors to deliver Tg cDNA in CBA/J mice [5,6]. This method has been shown to induce robust T cell proliferative and cytokine responses to Tg and its major T cell epitope (Tg.2098) [7], as well as higher titers of TgAb and anti-thyroperoxidase autoantibodies (TPOA) compared to control immunizations. The adenovirus-based model offers the advantage of achieving higher transgene expression in muscle tissue without the need for electroporation, making it less laborious.

One limitation of all these models is that they induce thyroiditis in wild-type mice expressing murine MHC class II molecules. Given the key role of MHC class II, specifically HLA-DR3, in triggering HT [8,9,10,11], there is a need for humanized mouse models of HT induced in mice expressing human HLA-DR3. One such model is induced by Tg plus adjuvant immunizations of NOD-DR3 mice. These mice are null for murine MHC class II and express human HLA-DR3. Here, we describe a novel murine model of HT using a different susceptible strain of mice, the CBA/J mice, that are null for murine MHC class II and express human HLA-DR3. This novel model can be very useful in studying the etiology of HT and in designing targeted immune therapies [12,13].

## 2. Materials and Methods

### 2.1. Generation of CBA/J-DR3 Mouse Strain Susceptible to EAT

The project was approved by the Albert Einstein College of Medicine Institutional Animal Care and Use Committee. We produced the CBA/J-DR3 mice by backcrossing C57BL/6J mice knockout for murine MHC class II and transgenic for human DRB1*0301 (C57BL/6J-DR3) to the wild-type CBA/J mice. The C57BL/6J-DR3 mice were originally generated by the team of G.J. Hämmerling [14]. Briefly, the DR3 transgenes (DRA1*0101/DRB1*0301) were inserted into F1 embryos of C57BL/6 X DBA/2, and the progeny were backcrossed to B10.Q mice. The DR3 mice were crossed with I-Eα/I-Aβ knockouts (C57BL/6-DR3 X 129/Sv) to obtain MHCII-knockout mice expressing human DR3 [9,15,16].

The CBA/J mouse strain was developed by Strong et al. [17] from a cross between a Bagg albino female and a DBA male mouse. It was introduced into the Jackson laboratory in 1948 and later into the Charles River Laboratories in 1982. This strain is widely used for different research purposes, especially in studies in immunology and inflammation. Moreover, this strain is susceptible to the development of experimental autoimmune thyroiditis (EAT) when immunized with thyroglobulin in adjuvant [18,19].

**Figure 1:**
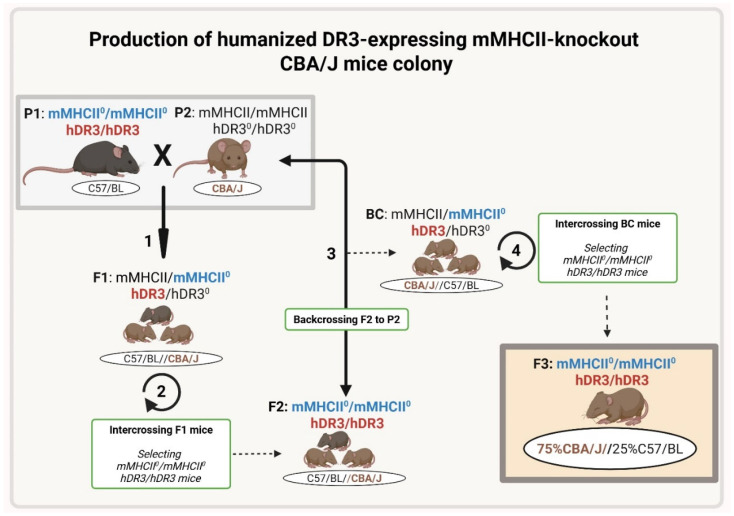
Production of a humanized human DR3-expressing mouse colony carrying 75% of the CBA/J strains’ genotype. (1) In the first step C57BL/6J-DR3 mice (P1) were backcrossed to wild-type CBA/J mice (P2) generating human-DR3/murine-MHCII (hDR3/mMHCII) C57/BL/6J-CBA/J heterozygous mice (F1). (2) Then, F1 mice were intercrossed and selected for the presence of the human HLA-DR3 gene and knockout for the murine MHC II genes, generating F2 mice that had 50% C57/BL background and 50% CBA/J background. (3) Then, F2 mice were backcrossed to CBA/J wild-type mice (P2) to generate the backcross generation (BC). (4) Furthermore, BC mice were intercrossed to generate hDR3/hDR3 homozygous mice (F3) that were knockout for the murine MHC II and had 75% CBA/J background and 25% C57/BL. The figure was created in Biorender. Aizhan Kozhakhmetova (2025) https://app.BioRender.com/illustrations/644a8ff0713e9321961b361c?slideId=e0e0a3a6-1541-4e5c-b029-6a2f27d182d8, accessed on 15 January 2026.

In the first step, we backcrossed C57BL/6J-DR3 mice to wild-type CBA/J mice, generating F1 mice that were C57/BL/6J-CBA/J heterozygotes carrying the human HLA-DR3 allele but also carrying the murine MHC II allele (from the CBA/J parents). We then intercrossed the F1 mice, selecting for those pups that were HLA-DR3 positive and knockout for the murine MHC II genes, generating F2 mice that had 50% C57/BL background and 50% CBA/J background. We then repeated the backcrossing with CBA/J wild-type mice and intercrossing of their pups to generate F3 mice that had 75% CBA/J background and 25% C57/BL and carried the human HLA-DR3 gene and were knockout for the murine MHC II genes. We confirmed that the F3 mice had 75% CBA/J background by performing a genome scan (performed by Jackson Laboratory, New York, NY, USA). Mice were bred in a pathogen-free animal facility (Albert Einstein College of Medicine, New York, NY, USA), and expression of HLA-DR3 was tested by PCR using DR3-specific primers to confirm the genotype: forward primer, 5′-CGCTTCGACAGCGAC-3′ and reverse primer 5′-GACAAATCCACACTCCAC-3′ (Figure 1).

### 2.2. Development of Mouse Model of EAT by Immunization with Thyroglobulin

CBA/J-DR3 mice generated as described above (female, 6–8 weeks of age) were injected subcutaneously into the inguinal area with 0.2 mg of Tg (Cell Sciences, Newburyport, MA, USA) in Complete Freund’s Adjuvant (CFA; Sigma-Aldrich, St. Louis, MO, USA) to induce classical EAT as previously described [2]. Briefly, mice were immunized with 0.2 mg Tg on day 0, boosted on day 7, and sacrificed on day 21. Control CBA/J-DR3 mice (female, 6–8 weeks of age) were injected with 100 μL phosphate-buffered saline (PBS) in CFA, following the same protocol, and sacrificed on day 21, too.

### 2.3. Splenocytes Isolation

Splenocytes were isolated from the dissected spleen as described before [6]. Briefly, mice spleens were harvested in complete RPMI 1640 (Corning, New York, NY, USA) supplemented with 10% fetal bovine serum (FBS) (Sigma-Aldrich, St. Louis, MO, USA) and 1 mM sodium pyruvate (Sigma-Aldrich, St. Louis, MO, USA). They were cut and pressed in a circular motion with a 10 mL syringe plunger, and the cell suspension was filtered through a 100-μm cell strainer twice and centrifuged at 200× *g* for 10 min at 4 °C. The supernatant was discarded, and the pellet was washed with RPMI followed by another centrifugation. To remove non-lymphocytic cells, 5 mL of Ammonium-Chloride-Potassium (ACK) lysis buffer was added to the cell pellet, and cells were incubated for 5 min at room temperature with intermittent shaking. Then 10 mL of complete RPMI 1640 was added and centrifuged at 200× *g* for 10 min. The supernatant was discarded, and the remaining pellet was resuspended in 3 mL of complete RPMI for counting and plating.

### 2.4. T-Cell Stimulation and Carboxyfluorescein Diacetate Succinimidyl Ester (CFSE) Analysis of Cell Proliferation

Splenocytes (2 × 10^6^ cells) were resuspended in 0.1% bovine serum albumin (BSA)/PBS and were labeled with 1.5 μM CFSE (Thermo Fisher Scientific, Waltham, MA, USA). After incubation for 10 min at 37 °C, the CFSE staining was terminated by the addition of 4 volumes of ice-cold complete RPMI 1640. After 5 min of incubation on ice, the cells were washed three times with fresh RPMI 1640 and resuspended in a fresh medium for counting and plating. The cells were treated with one of the following: medium alone, negative control (NC) peptide (GenScript, Piscataway, NJ, USA) (20 μg/mL), mouse CD3/CD28 beads (Thermo Fisher Scientific, Waltham, MA, USA) (as a positive control, 5 uL per 1 mlln of cells), Tg (Cell Sciences, Newburyport, MA, USA) (40 μg/mL), or Tg.2098 peptide (GenScript, Piscataway, NJ, USA) (20 μg/mL), known to be the major T-cell epitope in autoimmune thyroiditis [7] (Table 1). After 5 days of incubation at 37 °C in a 5%-CO_2_ incubator, cells were collected, and T cell proliferation was analyzed by flow cytometry and Flowjo 10.9.0 software (Tree Star, Ashland, OR, USA). Assays were performed in duplicates, and data are expressed as stimulation index (SI). We calculated SI by using the following formula: SI = [% proliferated lymphocytes (stimulant-treated)]/[% proliferated lymphocytes (non-treated)]. Proliferation index SI ≥ 1.5 was considered a positive response to the stimulant.

### 2.5. Cytokine Assay: Measuring Levels of Interferon-Gamma (IFN-γ), Interleukins IL-4, IL-10, and IL-17 in Splenocyte Supernatants Using Luminex 200

Mice splenocytes were stimulated with the same stimulants and concentrations used for the CFSE assay (see above), and incubated for 48 h at 37 °C in a 5%-CO_2_ incubator. After incubation, cell supernatant was collected and analyzed: IFN-γ, IL-4, IL-10, and IL-17 levels were measured by using a Milliplex mouse cytokines/chemokine magnetic panel (catalog no. MCYTOMAG-70K; EMD Millipore Corporation, Burlington, MA, USA) following the manufacturer’s instructions. Assays were read by using Luminex 200 with xPONENT 4.3 software (Luminex, Austin, TX, USA).

### 2.6. Anti-Thyroglobulin Antibody (TgAb) Measurement in Mice Sera Using Enzyme-Linked Immunosorbent Assay (ELISA)

Serum levels of TgAb were measured by enzyme-linked immunosorbent assay (ELISA) using sera from mice as previously described [6,12,20]. The signal was developed using freshly prepared para-nitrophenylphosphate substrate (Sigma-Aldrich, St. Louis, MO, USA), and the data are presented as absorbance (optical density at 405 nm).

### 2.7. Thyroid Stimulating Hormone (TSH) Measurement in Mice Sera Using Luminex 200

The concentrations of TSH in mouse sera were determined using a Milliplex mouse pituitary magnetic panel (MPTMAG-49K-01; EMD Millipore Corporation, Burlington, MA, USA) following the manufacturer’s instructions. Assays were read by using Luminex 200 with xPONENT software (Luminex, Austin, TX, USA).

### 2.8. Thyroid Histology

Mouse thyroids were removed at sacrifice and fixed in 10% neutral-buffered formalin and then embedded in paraffin. Sections of 4 μm thickness were prepared from the paraffin-embedded thyroids and stained with hematoxylin-eosin (H&E) by the Histology and Comparative Pathology Core at Albert Einstein College of Medicine. The images were acquired by using a 3DHistech Panoramic 250 Flash III slide scanner (3DHISTECH Ltd., Budapest, Hungary) at the Analytical Imaging Facility at Albert Einstein College of Medicine.

### 2.9. Thyroid Immunohistochemistry

Immunofluorescent immunohistochemistry (IHC) was performed on paraffin-embedded thyroid sections to visualize immune infiltrates (CD3^+^ T cells) in the thyroid. Sections were deparaffinized in xylene twice for 5 min, rehydrated through graded ethanol (100%, 90%, 80%, and 70%; 20 dips each), and washed twice in PBS for 10 min. Antigen retrieval was carried out using Target Retrieval Solution (DAKO; 1:10 in distilled water) in a Coplin jar placed in a pressure cooker at high pressure for 15 min, followed by rapid cooling and two 5 min PBS washes. Slide edges were dried, sections were circled with a Pap pen to create a hydrophobic barrier, and nonspecific binding was blocked with 10% BSA in 1× TRIS buffer containing 0.1% Triton for 30–45 min. Rabbit anti-CD3 primary antibody (1:200; Abcam, Waltham, MA, USA) was applied at 100 µL per section and incubated for 1 h at RT in a humid chamber, followed by three 5 min PBS washes. Goat anti-rabbit IgG Alexa Fluor 488 secondary antibody (1:500; Thermo Fisher Scientific, Waltham, MA, USA) was added at 200 µL per section and incubated for 30 min at RT in the dark, followed by three PBS washes. Nuclei were counterstained with DAPI (1 µg/mL in PBS) for 5 min in the dark, rinsed once in PBS, and sections were mounted with Fluoromount-G under a coverslip, dried, sealed, and imaged by fluorescence microscopy to assess T-cell distribution in thyroid tissue.

### 2.10. Statistical Analysis

SPSS Statistics 28 and GraphPad Prism 5 software were used to perform statistical analysis. Student’s *t*-test (unpaired or paired, two-tailed) for normally distributed and Wilcoxon signed rank (paired, two-tailed) or Mann–Whitney (unpaired, one-tailed) tests for skewed data were used for comparison of the experimental vs. control groups for each of the variables measured. Data is presented as mean ± standard deviation. A *p*-value < 0.05 or < 0.025 (corrected for two variables in between-group comparisons of cytokines) was considered statistically significant. No adjustment of *p*-value for multiple comparisons was applied to within-group cytokine analyses.

Group sizes (EAT *n* = 16, control *n* = 12) were selected based on prior EAT studies using similar readouts, in which cohorts of 8–12 mice per group provided >80% power to detect biologically relevant differences in T-cell proliferation and autoantibody levels [5].

### 2.11. Ethics

The project was approved by the Albert Einstein College of Medicine Institutional Animal Care and Use Committee (IACUC) on 11/19/2024 (protocol no. 00001023).

## 3. Results

### 3.1. T-Cell Proliferative Responses to Tg and Tg.2098 in CBA/J-DR3 Mice Immunized with Tg

**Figure 2:**
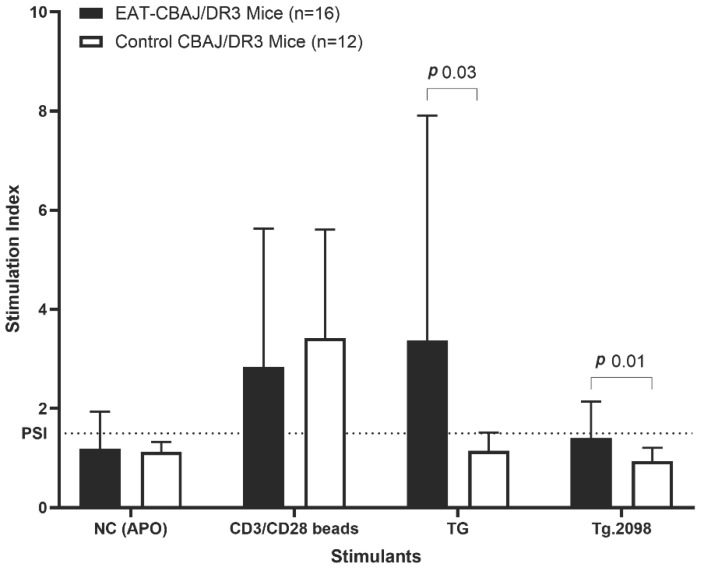
CFSE-based cell proliferation assay in splenocytes isolated from CBA/J-DR3 mice induced with EAT compared to Control-CBA/J-DR3 mice. EAT-CBA/J-DR3 mice immunized with human Tg developed strong T cell proliferative responses to Tg and Tg.2098, compared to control mice (*p* = 0.03 and 0.01, respectively). Stimulation indices (SI) of T cells stimulated with Tg and Tg.2098 from mice immunized with Tg (EAT mice, *n* = 16) and from mice injected with PBS/CFA (control mice, *n* = 12) are presented as mean ± SD; SI ≥ 1.5 was considered positive (dashed line is a threshold). Negative control (NC) peptide was used as a negative control, and stimulation with CD3/CD28 beads was used as a positive control. Mann–Whitney test, *p* value is one-tailed.

CBA/J-DR3 mice induced with EAT showed strong T-cell proliferative responses to both Tg and Tg.2098 peptide using the CFSE assay (Figure 2). The mean stimulation index (SI) calculated as described above was 3.4 ± 4.5 for Tg, and 1.5 ± 0.7 for Tg.2098. SI ≥ 1.5 was considered positive [7]. Control CBA/J-DR3 mice showed no T-cell responses to Tg or Tg.2098 (SI 1.1 ± 0.4 and 0.9 ± 0.3, respectively).

### 3.2. T-Cell Cytokine Responses to Tg and Tg.2098 in EAT-CBA/J-DR3 and Control-CBA/J-DR3 Mice

**Figure 3:**
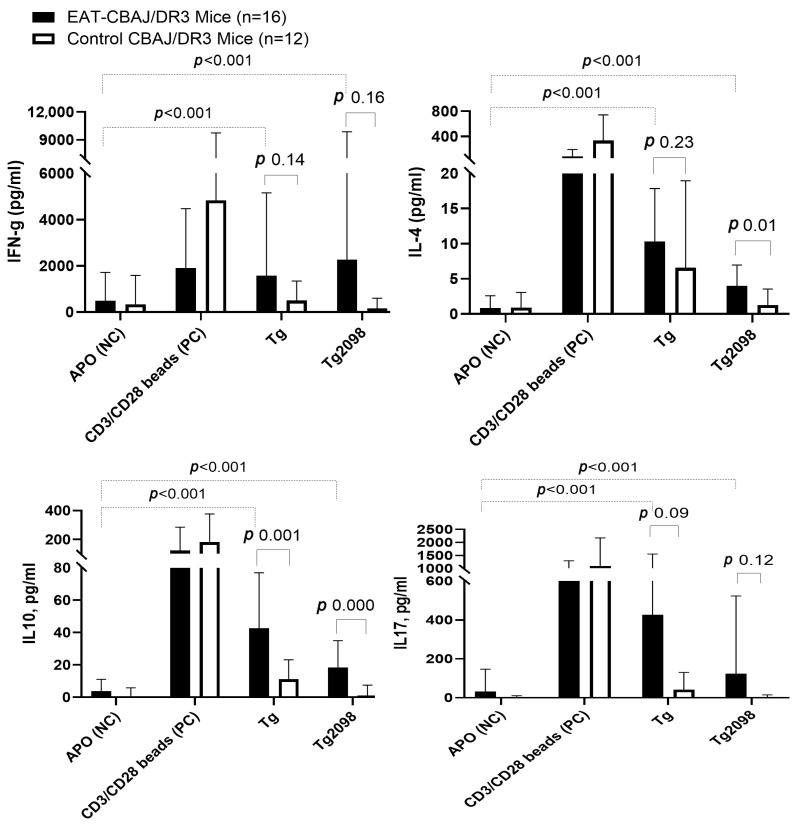
Cytokines IFN-γ, IL4, IL10, and IL17 levels in supernatants of splenocytes from EAT-CBA/J-DR3 and Control-CBA/J-DR3 mice stimulated with human Tg or Tg.2098 (Luminex analysis). In EAT-CBA/J-DR3 mice (*n* = 16) levels of IFN-γ, IL4, IL10, IL17 cytokines in supernatants of stimulated splenocytes tended to be higher than those in Control-CBA/J-DR3 mice (*n* = 12) but only reached statistical significance for IL4 and IL10 (*p* > 0.05 for IFNg and IL17; *p* < 0.01 for IL4 for Tg.2098 and IL10 for Tg and Tg.2098). Data are represented as means ± SD of cytokine levels in stimulated cells corrected to levels in unstimulated cells (medium) by subtraction. Statistical analysis was performed using a Mann–Whitney test. A one-tailed *p*-value < 0.05 or <0.025 (adjusted for multiple comparisons only for between-group analyses of cytokines) was considered statistically significant.

In EAT-CBA/J-DR3 mice (*n* = 16), strong T-cell responses to both Tg and Tg.2098 were observed as indicated by significantly higher levels of cytokines in supernatants of Tg- or Tg.2098-stimulated splenocytes (IFNg, 1577 ± 3580 pg/mL and 2266 ± 7600 pg/mL, respectively, for Tg and Tg.2098; IL4, 9.2 ± 8.4 pg/mL and 2.9 ± 3.7 pg/mL, respectively; IL10, 42.6 ± 34.3 pg/mL and 18.4 ± 16.6 pg/mL respectively; IL17 426.1 ± 1127 pg/mL and 123.7 ± 399.2 pg/mL respectively) compared to untreated splenocytes (*p* < 0.001) (Figure 3).

In Control-CBA/J-DR3 mice, which were not injected with the Tg antigen, no increase in cytokine production in cells stimulated with Tg or Tg.2098 was observed (*p* > 0.05) (Figure 3).

When directly comparing between mice groups, the levels of IFNg, IL4, IL10 and IL17 produced by stimulated splenocytes in EAT-CBA/J-DR3 mice tended to be higher than those in Control-CBA/J-DR3 mice; however, this difference was only statistically significant for IL4 (*p* 0.01 for Tg.2098) and IL10 (*p* 0.001 and 0.0005 for Tg and Tg.2098, respectively), but did not reach statistical significance for IFNg (*p* = 0.14, and *p* = 0.16 for Tg and Tg.2098, respectively) and IL17 (*p* = 0.09, and *p* = 0.12 for Tg and Tg.2098, respectively), likely due to the high variability of cytokine levels within each group (Figure 3).

### 3.3. Anti-Thyroglobulin Antibodies (TgAb) in Sera of CBA/J-DR3 Mice Induced with EAT

**Figure 4:**
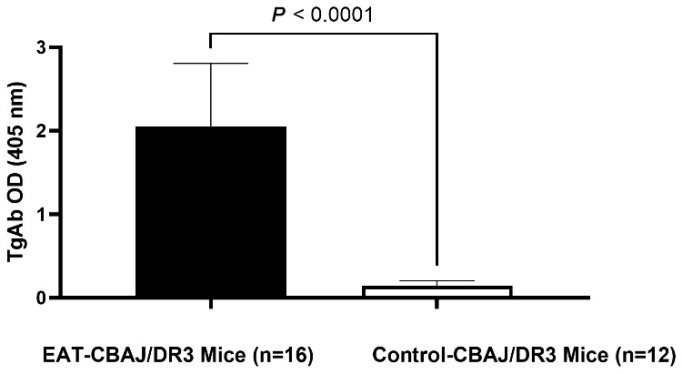
Anti-thyroglobulin antibodies (TgAb) levels in sera of EAT-CBA/J-DR3 and Control-CBA/J-DR3 mice. CBA/J-DR3 mice (EAT mice, *n* = 16) immunized with Tg showed high levels of anti-Tg antibodies 21 days after initiation of the immunization protocol. Control mice (*n* = 12) injected with PBS/CFA did not develop antibodies. Data are represented as means ± SD. Statistical analysis was performed using an unpaired *t*-test. A two-tailed *p*-value < 0.05 was considered statistically significant.

EAT-CBA/J-DR3 mice had significantly higher levels of thyroglobulin antibodies (2.05 ± 0.75) when compared to control mice (0.15 ± 0.06) (*p* < 0.0001) (Figure 4).

### 3.4. Thyroid-Stimulating Hormone (TSH) in CBA/J-DR3 Mice Sera

**Figure 5:**
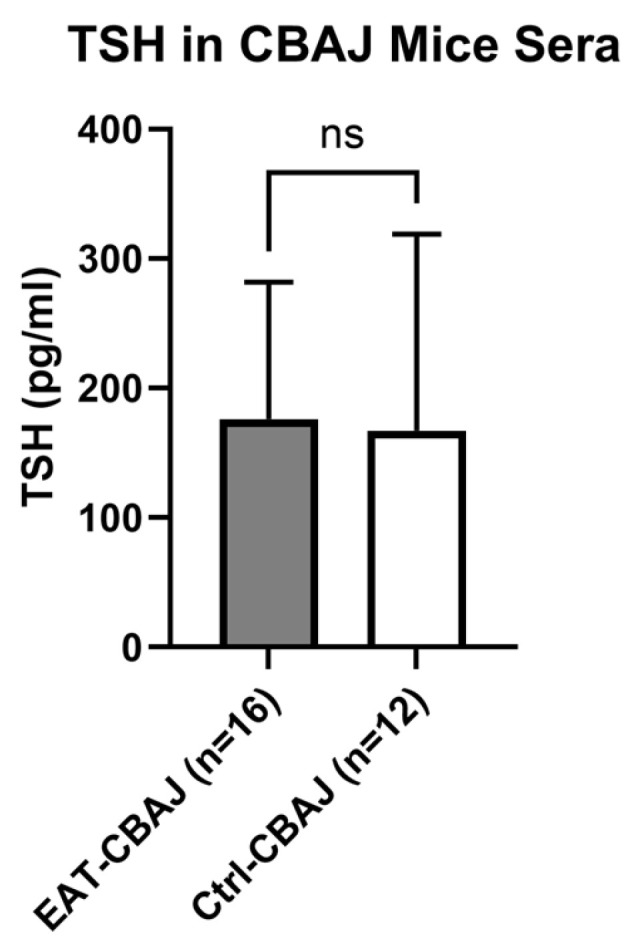
Thyroid-stimulating hormone (TSH) level in sera of EAT-CBA/J-DR3 and Control-CBA/J-DR3 mice (Luminex). The level of TSH in EAT-CBA/J-DR3 mice (*n* = 16) was not significantly different from that in control-CBA/J-DR3 mice (*n* = 12). Data are represented as mean ± SD. Statistical analysis was performed using an unpaired *t*-test. A two-tailed *p*-value < 0.05 was considered statistically significant, ns—non-significant.

TSH levels in mouse sera were not significantly different when comparing EAT-CBA/J-DR3 mice (mean ± SD, 176.3 ± 105 pg/mL) and Control-CBA/J-DR3 mice (167.3 ± 151 pg/mL) (*p* = 0.81) (Figure 5).

### 3.5. Thyroid Histology and Immunohistochemistry

**Figure 6:**
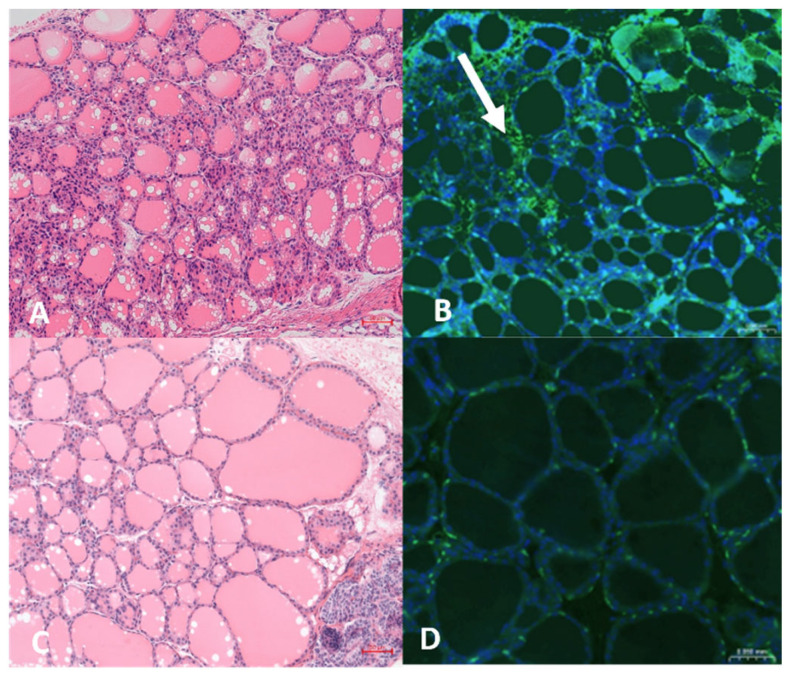
Hematoxylin and eosin-stained (H&E) sections (**A**,**C**) and immunostaining of CD3^+^ T-cells (**B**,**D**) of thyroid gland of EAT-CBA/J-DR3 (**A**,**B**) and Control-CBA/J-DR3 (**C**,**D**) mice. In the H&E-stained thyroid section of an EAT-CBA/J-DR3 mouse, no clear lymphocytic infiltration was detected in Tg immunized mice (**A**); however, on immunostaining, minimal infiltration with CD3^+^ T-cells was detected in Tg immunized mice (shown with white arrow) (**B**). No lymphocytic infiltration was seen in the control mice (**C**,**D**). Images were taken using a Leica confocal microscope (20×) (Leica Microsystems GmbH, Wetzlar, Germany), Panoramic 250 Flash III slide scanner was utilized to generate images. Green, AF488 CD3^+^ T-cells staining; Blue, DAPI staining for nuclei.

On H&E staining, the thyroids of Tg-immunized mice (Figure 6A) did not show lymphocytic infiltration; however, immunohistochemistry showed minimal infiltration with CD3-positive T-cells (Figure 6B). No changes were observed in control mice injected with PBS/CFA (Figure 6A,C).

## 4. Discussion

Mouse models of AITD are a useful tool to dissect the mechanisms causing AITD and to develop novel therapies. Given that HLA-DR3 has been shown to be the major susceptibility gene for AITD, and that the mechanism is believed to be through presentation of immunogenic thyroglobulin peptides to T-cells [21], developing humanized mouse models of AITD using mice that express HLA-DR3 is especially useful. Our novel EAT mouse model in CBA/J-DR3 mice demonstrates key features of human autoimmune (Hashimoto’s) thyroiditis, including T-cell and antibody responses to Tg and the pathogenic Tg.2098 peptide.

Indeed, these responses included both proliferative T-cell activation and enhanced cytokine production upon Tg/Tg.2098 stimulation. Furthermore, EAT CBA/J mice developed high titers of anti-thyroglobulin antibodies and minimal CD3^+^ T-cell infiltration of the thyroid, which are hallmark features in the diagnosis of autoimmune thyroiditis in humans. The development of T cells reactive to thyroid autoantigens and elevated anti-thyroid antibodies suggests a breakdown of immune tolerance to thyroid self-antigens, mirroring the pathogenesis of human autoimmune thyroiditis.

Compared to previously published EAT models, the immunologic profile observed in the CBA/J-DR3 mice aligns well with earlier HLA-DR3 transgenic systems, demonstrating a similar autoimmune profile. Similar to DR3 transgenic NOD and B10 backgrounds, in which human or mouse Tg immunization induces robust T-cell proliferation to Tg epitopes and thyroiditis, our CBA/J-DR3 mice developed strong proliferative responses to human Tg and as well as to the immunodominant human epitope Tg.2098, elevated anti-Tg antibody titers, and thyroidal T-cell infiltration, confirming that DR3 is sufficient to confer susceptibility to Tg-driven thyroid autoimmunity. Thus, our data confirm the key role of HLA-DR3 in the development of autoimmune thyroiditis, as autoimmune thyroiditis could be induced in HLA-DR3 mice of two different autoimmune-prone strains (NOD and CBA/J). Moreover, our model validated the key role of Tg.2098 as the major T-cell epitope in EAT induced in humanized HLA-DR3 mice by human Tg, and by extension in human autoimmune thyroiditis. A major advantage of our model is that the NOD-DR3 strain is very sensitive and difficult to breed and maintain, while the CBA/J-DR3 strain is much more robust and easier to maintain [2,9].

The observed T-cell reactivity and cytokine responses highlight the central role of T-cell-mediated autoimmunity in thyroid disease. We have intentionally selected these cytokines due to their distinct roles in immune regulation: IFN-γ, a key Th1 cytokine, and IL-17, the key Th17 cytokine, contribute to thyroid inflammation by activating macrophages and NF-κB-mediated signaling, while IL-4 is a key Th2 response cytokine, and IL-10 exerts counter-regulatory effects that suppress T-cell responses, thereby modulating the severity and progression of autoimmune thyroiditis [22,23,24,25,26,27]. The balance among these cytokines is critical in the pathogenesis of autoimmune thyroiditis, influencing disease severity and progression. The high variability seen between mice in cytokine levels is typical for EAT models and is likely the result of variations in individual animals’ T cell responses, which can be caused by genetic and epigenetic factors, as well as uneven antigen exposure during immunization, and variability in Tg-specific memory T-cells activation. This variation mirrors the immune diversity present in humans [26]. Our findings are consistent with previous EAT models and further substantiate the importance of T cell-driven mechanisms of autoimmunity in AITD [5,20]. Recent studies in HLA-DR3 transgenic mice have demonstrated that antigen-specific peptide therapies targeting thyroid epitopes can induce regulatory T-cells and prevent disease progression, highlighting the therapeutic potential of modulating DR3-restricted responses [28].

We did not observe significant differences in TSH levels in our EAT mouse model compared to control mice; however, TSH levels may not dramatically change within 3 weeks because the autoimmune response and thyroid tissue destruction require more time to induce hypothyroidism and elevate TSH levels. Early phases of autoimmune thyroiditis often involve immune cell infiltration and follicular damage without immediate hypothyroidism, which typically develops over a longer period, as seen in studies where TSH elevation occurs after several weeks or months of sustained thyroid damage [5,29]. In line with this early disease stage, thyroid histology at day 21 showed only minimal CD3^+^ T-cell infiltration on immunohistochemistry, which is less extensive than the dense lymphocytic infiltrates typically reported in classical Tg/CFA-induced EAT models examined at later time points or after repeated immunizations [2].

Although this model has clear strengths, several limitations should be acknowledged and addressed in future work. First, thyroidal inflammation in CBA/J-DR3 mice was very mild at the 3-week time point, with only minimal CD3^+^ T-cell infiltration and no clear changes in TSH, suggesting that the current immunization protocol primarily captures early immunologic events rather than fully established destructive thyroiditis; extending follow-up, varying antigen dose/adjuvant, or adding booster immunizations may be necessary to model later stages of HT with overt hypothyroidism and more pronounced histologic damage. Second, only female mice and a single-strain background were studied, which reflects the female predominance and HLA-DR3 association in human AITD but may limit generalizability; including males and additional HLA or strain combinations in parallel experiments would help clarify sex- and background-dependent effects on disease expression. Finally, while T-cell proliferation, cytokine production, and TgAb responses were characterized, other relevant immune compartments such as B-cell subsets, regulatory T cells, and innate immune cells were not assessed in this study, and future studies incorporating detailed immunophenotyping and functional assays will be important to verify the full cellular network driving disease in this model [13,30].

## 5. Conclusions

The use of humanized CBA/J-DR3 mice, which express human HLA-DR3, is a significant strength of our model. HLA-DR3 has been associated with increased susceptibility to AITD in humans, and its expression in these mice enhances the translational relevance of this new model [20,30]. Unlike some other EAT-susceptible strains, the novel CBA/J-DR3 mice offer new insights into HLA-DR-specific aspects of disease induction and progression. It is important to note that using the CBA/J-DR3 mouse strain to develop the EAT model has several benefits over utilizing the transgenic C57BL/6J-DR3 parent mouse strain itself. It was found that the C57BL/6J-DR3 mouse strain develops a strong Th2-biased inflammation, while the CBA/J-DR3 mice exhibit a less pronounced type 2 inflammatory response [31]. Furthermore, CBA/J mice are resistant to developing atherosclerosis (which is a chronic inflammatory condition that can exacerbate autoimmune conditions and bias outcomes of EAT studies), and the wild-type CBA/J mouse strain is frequently used to study granulomatous EAT [32]. This genetic background may allow for more accurate testing of potential therapeutic interventions targeting HLA-DR3-mediated autoimmune processes. Given its human HLA-DR3 background and robust responses to human Tg and Tg.2098, this model is particularly well suited for testing antigen-specific interventions such as tolerogenic Tg- or Tg.2098-derived peptides and retro-inverso peptides or small molecules that block DR3-restricted antigen presentation. In addition, CBA/J-DR3 mice could be used to evaluate broader immune-modulating strategies relevant to AITD, including agents that enhance regulatory T-cell function or checkpoint-based approaches that regulate pathogenic T-cell responses. Additionally, this model could be valuable for investigating the interplay between genetic susceptibility (conferred by HLA-DR3) and environmental factors in the development of autoimmune thyroiditis. To sum up, this model has the potential to accelerate the development of more targeted and effective treatments for AITD.

## Figures and Tables

**Table 1:** Amino acid sequences of peptides used for T-cell stimulation in the cell proliferation assay.

Peptide	Amino Acid Sequence
Tg.2098	LSSVVVDPSIRHFDV
Negative control	PKDRLKIYNNFTKIGDLSL

## Data Availability

The raw data supporting the conclusions of this article will be made available by the authors on request.

## Abbreviations

The following abbreviations are used in this manuscript:

ACK	Ammonium-chloride-potassium
AITD	Autoimmune thyroid diseases
BSA	Bovine serum albumin
CFA	Complete Freund’s adjuvant
CFSE	Carboxyfluorescein diacetate succinimidyl ester
EAT	Experimental autoimmune thyroiditis
ELISA	Enzyme-linked immunosorbent assay
FBS	Fetal bovine albumin
H&E	Hematoxylin-eosin
HT	Hashimoto’s thyroiditis
IACUC	Institutional Animal Care and Use Committee
IFN-γ	Interferon-gamma
IL	Interleukin
NC	Negative control
PBS	Phosphate-buffered saline
SD	Standard deviation
SI	Stimulation index
Tg	Thyroglobulin
TgAb	Anti-thyroglobulin antibody
TPOA	Anti-thyroperoxidase autoantibodies
TSH	Thyroid-stimulating hormone
